# 2013 and ‘DSMitis’: implications for the *Journal of Eating Disorders* and its authors

**DOI:** 10.1186/2050-2974-2-3

**Published:** 2014-01-22

**Authors:** Phillipa Hay, Stephen Touyz

**Affiliations:** 1 School of Medicine, Centre for Health Research, University of Western Sydney, Sydney, Australia; 2 School of Medicine, James Cook University, Townsville, Australia; 3 Schools of Psychology and and Centre for Eating and Dieting Disorders (Boden Institute), University of Sydney, Sydney, Australia

## Editorial

Some of us can recall the fervour and excitement surrounding the introduction of the third revision of the American Psychiatric Association’s Diagnostic and Statistical Manual of Mental Disorders (DSM) [1] onto the international psychiatric stage in 1980. History will judge the fifth revision of the DSM (DSM-5) [2] and whilst the response thus far appears more muted there are a number of substantive changes already impacting on clinicians and academics. For example, lecturers revising their power-points find it a much simpler system to explain with no axes to distinguish it from general medical classifications or to be a proxy for a simplified formulation. In assessment, Engel [3] and colleagues’ ‘biopsychosocial’ approach to psychiatric formulation may thus resume importance in capturing the understanding of the patient’s problems beyond the diagnosis. Whether the WHO disability scale [4] will have the utility and familiarity as the Global Assessment of Function (GAF [5]) is also unknown, particularly given the highly variable use of the GAF.

Beyond the clinician what do the changes mean for academics? Already instruments like the Mini International Neuropsychiatric Interview [6] need urgent official upgrades. Fortunately the gold standard assessment instrument in Eating Disorders research, the Eating Disorder Examination (EDE) [7] and its self-report sister instrument the EDE-Q [8] require little adaptation and indeed have benefited from the alignment of criteria for presence of bulimia behaviours across disorders. Thus researchers will no longer have to inquire about binge eating over the past 6-months (as it is now diagnosed over 3-months) and the final question on menstrual function is no more a diagnostic item for anorexia nervosa.

Will *Journal of Eating Disorders* accept papers using DSM-IV [5] criteria? The short answer is in most instances ‘no’, unless authors make a compelling argument. For example in a longitudinal study it is usually not possible to reclassify participants diagnosed according to DSM-IV criteria at entry to the study. There will be an expectation that authors doing systematic reviews carefully check the criteria used for diagnoses in papers. Many applied broad criteria for bulimia nervosa or anorexia nervosa (e.g., the ‘Oxford’ criteria of once weekly binge eating [7]) that now accord with DSM-5 criteria and this should be reported in the review.

What about the alternate international scheme, the World Health Organisation’s International Classification of Diseases and Related Health Problems (ICD)? The tenth revision of the ICD (ICD-10) and DSM-IV schemes currently use similar diagnostic terms and the same numerical systems. This assisted hospital administrators who need to convert DSM-IV into ICD-10 for government data collection and similar purposes, but it was of less interest to researchers and academics. Now however there is potential for confusion with the DSM-5 using some ICD-10 terms, e.g., ‘atypical’ anorexia nervosa and bulimia nervosa, with different criteria. In addition, the 11th revision of the ICD [9] may remove the requirement of an objectively large amount in the criterion for binge eating episodes. These changes are of notable concern to researchers attempting to estimate incidence and prevalence data across time and space. With loosening of some diagnostic criteria (e.g., removal of the amenorrhoea criteria for anorexia nervosa) and expansion of the field to new and added disorders of feeding and eating (e.g., Avoidant/Restrictive Food Intake Disorder (ARFID)), care will need to be taken to avoid artificial increases in estimates of burden.

And looking forward? We anticipate a stream of papers with reference to the new eating disorders, in particular ARFID. This is both from the child and adolescent and the adult fields as ARFID encompasses both. The area is wide open for research on ARFID assessment, epidemiology, treatment and outcome. Such manuscripts will be particularly welcome and a special supplement is under consideration.

A major shift in conception is that body image concerns are no longer a feature of several feeding and eating disorders. These include ARFID and night eating syndrome, and the most common single disorder, binge eating disorder. We can expect new research investigating the validity of such eating disorders without weight and shape concerns as well as disorders not currently in the feeding and eating disorder grouping, but with such concerns, e.g. body dysmorphic disorder and muscle dysmorphia [10]. Debate over the interface with obesity will continue as well to stimulate scholarship. In the spirit of ‘closing the gap’ between the two fields *Journal of Eating Disorders* will continue to welcome relevant papers on weight disorders.

We reassure those of you suffering from “DSMitis” that the *Journal* will remain true to our mission of publishing primary research that broadly advances our understanding of the disorders seen and treated in clinics across the globe. In its first 12 months *Journal of Eating Disorders* received over 50 submissions, published its first supplement and over 40 papers including 29 research papers, nine reviews, four letters, commentaries/editorials and two book reviews, and awarded two prizes. In addition to counting numbers a qualitative appraisal shows a diversity of publication content from the fields of body image [11] to psychopharmacology [12]. We have had three press releases and are building an active social presence and community around the journal via Twitter, Facebook and Google+. The introduction of a 100 word lay summary we regard as an important development for dissemination of research findings beyond the academic world. In an era of information overload its visibility beneath the titles we anticipate will also guide readers in their decision whether or not to inspect the article further.

It is hard to believe that we are past our first anniversary! We take the opportunity to thank our Editorial Board, Associate Editors and Managing Editor, Mr Jeremy Freeman, Sara Ho and the wonderfully supportive staff at BioMed Central, and especial thanks to our authors whose scholarship ensured the early success of the Journal. All our best wishes to you and our readers for 2014!

Phillipa Hay and Stephen Touyz, Editors in Chief, January 2014.
